# Elevated levels of sCD48 are inversely correlated with markers of disease activity in bullous pemphigoid

**DOI:** 10.1111/exd.14679

**Published:** 2022-10-05

**Authors:** Emma Treco, Eunice Huan, Afshin Varzavand, Janet A. Fairley, Kelly N. Messingham

**Affiliations:** ^1^ Department of Dermatology University of Iowa Iowa City Iowa USA

**Keywords:** autoantibody, autoimmune, eosinophil, IgE, skin

## Abstract

sCD48 is elevated in diseases characterized by IgE and eosinophilia. Thus, serum levels sCD48 were evaluated in relation to clinical characteristics of Bullous pemphigoid (BP) patients. sCD48 levels were determined by ELISA in sera from 26 patients with classic BP and 26 healthy controls. Disease severity scores, differential blood counts, and circulating autoantibody levels were obtained. A correlation analysis was performed to establish relationships between sCD48 and clinical and laboratory markers of disease severity. Overall, circulating levels of sCD48 were significantly elevated in BP patients; however, when stratified based on disease severity, patients with mild–moderate disease had higher levels of sCD48 than those with severe disease. A Spearman's correlation analysis identified an inverse relationship between sCD48 and disease activity, serum BP180 IgE and peripheral eosinophil numbers. Further studies are needed to determine the pathologic relevance of these findings.

## 1 INTRODUCTION

Bullous pemphigoid (BP) is a chronic autoimmune blistering disease of the elderly that is characterized by urticarial or eczematous lesions and the formation of tense, fluid‐filled blisters.^1^ Histologic examination of BP lesions reveals separation at the dermal–epidermal junction and an inflammatory infiltrate comprised of eosinophils, accompanied by lymphocytes, mast cells and neutrophils.^1^, ^2^, ^3^ Pathogenesis is mediated primarily by immune and inflammatory events initiated by both IgG and IgE autoantibodies targeting BP180 (Collagen XVII), a hemidesmosomal protein involved in epidermal adhesion.^4^, ^5^ Standard therapy includes treatment with high dose steroids, often in combination with adjuvant immunosuppressive or immunomodulatory therapy, which leads to significant morbidity.^6^

CD48 is a costimulatory immunoglobulin‐like receptor that is found on the surface of haematopoietic cells and as a circulating soluble (sCD48) receptor.^7^, ^8^ Expression of both receptor forms is modulated by inflammation and they play a key role in the interaction of mast cells and eosinophils.^9^, ^10^, ^11^ In studies of asthma, serum sCD48 varies with disease severity, suggesting that it may be useful as a non‐invasive biomarker for diagnosis and/or therapeutic guidance.^7^, ^8^, ^12^ Given the common association of IgE and eosinophils in both asthma and BP,^5^, ^13^, ^14^, ^15^ the goal of this study was to evaluate sCD48 as a potential biomarker or regulator of the inflammatory milieu in BP.

### 1.1 Question addressed

Are circulating levels of sCD48 modulated in BP and do these levels correlate with other clinical or laboratory indicators of disease activity?

## 2 METHODS

### 2.1 Ethics statement

This study was approved by the University of Iowa Institutional Review Board (IRB#201607748), performed in accordance with the Declaration of Helsinki. Written informed consent was obtained prior to enrolment.

### 2.2 Study population and samples

This is a retrospective study utilizing banked sera and blister fluid stored from patients with a new onset of classic BP at the Department of Dermatology at the University of Iowa Hospitals & Clinics. Inclusion criteria were a clinical picture consistent with BP, subepidermal blistering with autoantibody reactivity to the epidermal side of salt split skin, and detection of circulating BP180 or BP230 IgG by ELISA. Healthy controls were patients on routine visits to the clinic who had no history of autoimmunity. Peripheral blood was drawn, and blister fluid (if available) was obtained by sterile aspiration. Sera and clarified blister fluid were stored at −80°C until use. Disease activity scores (BP Disease Area Index [BPDAI^16^]), medications, and circulating complete blood counts (CBC) were obtained from the patient record.

### 2.3 ELISA

IgG autoantibodies (BP180, RG‐M7613‐D; BP230, RG‐M7612‐D, MBL International, Japan) and levels of sCD48 (ABIN6965593, Antibodies‐online) were evaluated as directed using commercially available ELISA kits. BP180‐specific IgE was evaluated using a previously described ELISA.^13^ Both BP180 ELISAs evaluated reactivity to the immunodominant NC16A domain of BP180. Total IgE (normal 1.5‐144 IU/ml^17^) was evaluated using electrochemiluminescence at the University of Iowa Diagnostic Laboratories.

### 2.4 Analysis

Statistical analysis was performed using GraphPad Prism 9.0. Due to their abnormal distribution, antibody and sCD48 levels are expressed as median and interquartile range (IQR), defined as the difference between the 1st and 3rd quartile values. Comparisons between two groups were made using an unpaired two‐tailed Mann–Whitney test and Spearman's r was used to perform a correlation analysis. In all cases, a significance level was set at α = 0.05.

## 3 RESULTS

**FIGURE 1:**
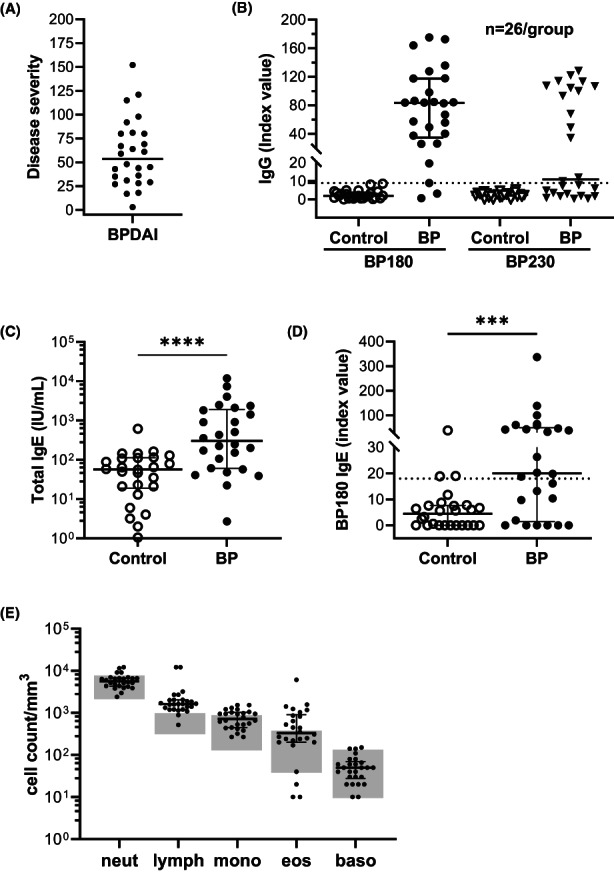
Characterization of BP patients. Twenty‐six patients with new onset of classic BP were included in this study. (A) Disease activity was scored using the Bullous Disease Area Index (BPDAI). (B) Serum IgG specific for BP180 and BP230 was measured by ELISA (dotted line equals cut‐off for a positive test, >9). (C) Serum IgE was measured using electrochemiluminescence and (D) IgE specific for BP180 was measured by ELISA (dotted line equals cut‐off for a positive test, >18). (E) Circulating complete blood counts for BP patients; the shaded area indicates the normal adult range for each cell type. The error bar indicates median ± interquartile range. ***p = 0.0080 ****p = 0.0001, Mann–Whitney test

This study included 26 new‐onset BP patients (14 male/12 female, mean age [±SD] 72.6 ± 13.6 years) and 26 healthy controls, typically seen for skin checks, with no history of autoimmunity (14 male/12 female, 75.2 ± 8.0 years). Disease activity scores ranged from 3–152 (median score 53.5) with half (13/26) presenting with severe BP (BPDAI ≥57^18^) (Figure 1A).

Evaluation of antibody levels in BP sera by ELISA revealed that 24/26 (92%) were positive for BP180‐specific IgG and 14/26 (54%) had BP230‐specific IgG (Figure 1B). The two patients who were negative on the BP180 IgG ELISA (<9.0 Index Value), were highly positive for BP230 IgG (>65 Index Value). Total IgE was significantly elevated in BP sera (Figure 1C, *p* = 0.0001) as was BP180‐specific IgE, with 14/26 (54%) positive sera (Figure 1D, *p* = 0.008). Due to the retrospective nature of this study, CBC information was available from all 26 BP patients but only 2 controls (not shown), so cell numbers for the BP patients are shown in relation to the normal adult reference range (University of Iowa Diagnostic Laboratory). Using this approach, nearly all BP patients had elevated lymphocyte numbers, many had elevated eosinophil and monocyte counts, but very few had elevated neutrophil or basophil counts (Figure 1E).

**FIGURE 2:**
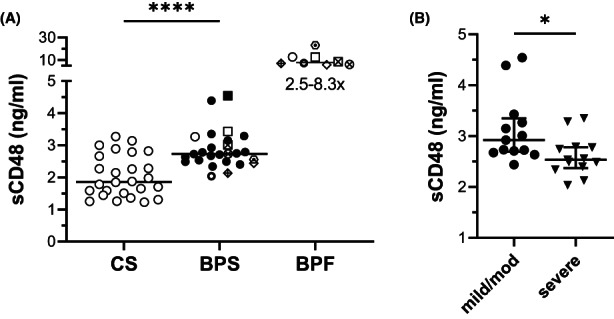
Serum levels of sCD48 are elevated in BP. sCD48 was measured by ELISA. (A) sCD48 levels in sera from 26 BP patients (BPS) and 26 controls (CS), and in fluid aspirated from blisters of a subset (*n* = 8, BPF) of BP patients. Matched symbols indicate the BPS and its corresponding BPF from the same patient. (A) The level of sCD48 was significantly elevated in BP versus control sera (****p < 0.0001, Mann–Whitney test). Comparison of serum and blister fluid from the same patient (matched symbols) revealed a 2.5–8.3‐fold increase in sCD48. (B) sCD48 levels were higher in patients with mild–moderate (mild‐mod) verses severe BP, using BPDAI >57 as an indicator of severe disease (*p = 0.0295, Mann–Whitney test). The error bar indicates median ± interquartile range

sCD48 was evaluated in sera from BP patients and controls, along with the corresponding blister fluid, which were available from a subset (*n* = 8) these patients. Overall, sCD48 levels were significantly elevated in BP sera compared with controls (*p* < 0.0001). Moreover, when serum and blister fluid from the same patient were compared, sCD48 was 2.5–8.3× higher in blister fluid (Figure 2A). Interestingly, when BP patients were grouped based on disease severity, serum levels of sCD48 were higher in patients with mild to moderate disease (BPDAI ≤57) compared with patients with severe disease (BPDAI >57) (median ± 95%CI; 2.922 ± 0.656 vs. 2.535 ± 0.430, *p* = 0.0295) (Figure 2B).

**FIGURE 3:**
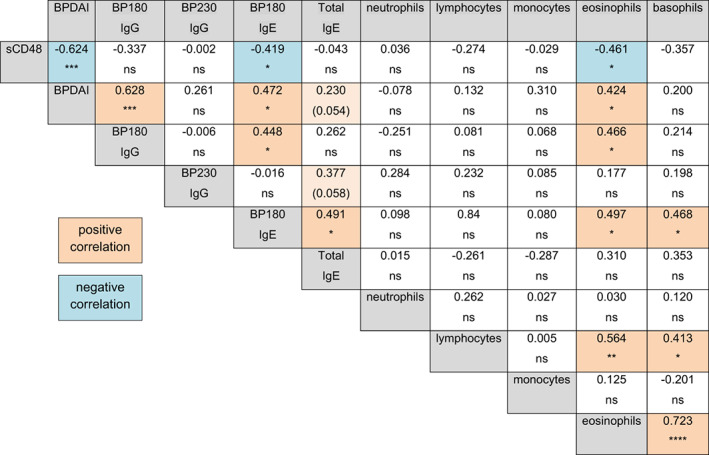
sCD48 levels are inversely correlated with hallmarks of BP activity. A Spearman's correlation analysis was performed to determine the strength of association (r) and level of significance (*p*‐value) between sCD48 levels and other characteristics of BP. Significant positive (orange) and negative (blue) correlations are highlighted. *p ≤ 0.05, **p ≤ 0.01, ***p ≤ 0.001, ****p ≤ 0.0001, ns = not significant

A Spearman's correlation was used to identify relationships between sCD48 and these other characteristics of BP patients; positive correlations are shaded orange and negative correlations are shaded blue (Figure 3). As expected, BPDAI scores were closely related to BP180‐specific IgG (*r* = 0.628, *p* < 0.0001) and IgE (*r* = 0.472, *p* < 0.05) levels and circulating eosinophil numbers (*r* = 0.424, *p* < 0.05).^5^, ^14^, ^15^ BP180 IgG was associated with BP180 IgE (*r* = 0.448, *p* < 0.05) and eosinophil numbers (*r* = 0.466, *p* < 0.05). Additionally, BP180 IgE levels were directly associated with circulating eosinophil and basophil counts and eosinophil and basophil counts were directly related (*r* = 0.723, *p* < 0.0001). Examination of relationships between sCD48 and these factors revealed a strong inverse correlation with BPDAI scores (*r* = −0.624, *p* < 0.001), BP180 IgE (*r* = −0.419, *p* < 0.05) and circulating eosinophil numbers (*r* = −461, *p* < 0.05), but no relationship with IgG autoantibody levels or total IgE. Additionally, the strong inverse relationship between sCD48 and BPDAI was maintained in subsets of BP patients (Table S1), such as those with IgG antibodies targeting both BP180 and BP230 (*r* = −0.666, *p* < 0.05) or those with elevated total (*r* = −0.670, *p* < 0.001) or BP180‐specific IgE (*r* = −0.670, *p* < 0.05).

## 4 DISCUSSION

This study identified an overall increase in serum sCD48 in BP patients with levels that correlated inversely with several established hallmarks of disease activity, including BPDAI scores, BP180‐specific IgE antibody levels, and circulating eosinophil numbers. These findings are similar to those in asthma, where sCD48 levels are highest in patients with mild disease but decline with increasing disease severity.^12^, ^19^ The marked difference in sCD48 levels between patients with mild asthma and healthy controls, led to its proposal as a sensitive yet non‐invasive diagnostic biomarker.^12^ In BP, disease severity has been evaluated in relation to a wide array of serum factors, including cytokines, chemokines and soluble receptors in search of reliable disease biomarkers, therapeutic targets and predictors of therapeutic response.^5^, ^14^ To date, the best‐established marker of disease activity is serum level of BP180‐specific IgG, although levels do not always correlate with disease activity.^1^, ^20^ Severity of BP has also been associated with circulating IgE, eosinophil numbers or factors involved in the recruitment and activation of eosinophils.^5^, ^14^, ^15^

In our cohort, BPDAI was directly associated with levels of BP180 IgE and circulating eosinophil numbers, as expected, and was inversely associated with sCD48. Since BP180 IgG is one of the most reliable indicators of disease activity, it interesting that the strength of association between sCD48 and BPDAI is similar to what is observed between BP180 IgG and BPDAI, but no significant correlation was observed between sCD48 and BP180 IgG. Evaluation of sCD48 at distinct phases of disease and during clinical therapy will be necessary to understand its relevance to disease or its utility as a diagnostic marker of BP.

It is possible that sCD48 is relevant in certain patient subsets based on autoantibody profiles or the degree of eosinophilia. Since IgE is thought to play a key role in the activation of mast cells and eosinophils in BP, the inverse association of sCD48 with these factors suggests that it may be related to IgE‐mediated mechanisms of BP. It is also possible that sCD48 levels do not play a direct role in BP pathogenesis but serve as an indicator of the inflammatory response. A major shortcoming is the lack of CBC data in healthy controls which is needed to evaluate the relationship between sCD48 and different cell subsets without disease. To better understand the relevance of these findings to BP, a prospective study should also include patients with other cutaneous diseases not characterized by IgE.

A limitation is the absence of studies evaluating cutaneous expression of CD48. Through interaction with its ligand, 2B4, CD48 is a primary mediator of the bidirectional interaction between mast cells and eosinophils.^21^, ^22^, ^23^ In vitro evidence suggests that sCD48 interaction with CD244 effectively blocks this interaction, resulting in decreased cellular activation and a decreased release of inflammatory mediators.^21^, ^24^ Since the sCD48 levels were much higher in blister fluid, when compared to sera, it is likely that the soluble isoform, created by cleavage from the membrane, is generated in the skin.^8^ Thus, we hypothesize that CD48 plays a role in activation of cutaneous mast cells and eosinophils in BP and that the soluble isoform provides a feedback loop to limit inflammation. For some unknown reason, the role of sCD48 is diminished as disease progresses (higher BPDAI). This could reflect the level of participation of cutaneous mast cells and eosinophils or IgE early in disease.^25^, ^26^, ^27^, ^28^ Evaluation of sCD48 expression in BP lesions is necessary to explore this hypothesis.

## AUTHOR CONTRIBUTIONS

Study conception and design: KNM. Acquisition of Data: ET, EH, AV, JAF, KNM. Analysis and interpretation of data: ET, EH, AV, KNM. Drafting and/or critical review of the manuscript: ET, EU, KNM. All authors have read and approved the final manuscript.

## CONFLICT OF INTEREST

The authors have declared no conflicts of interest.

## Supporting information

Table S1

## Data Availability

The data that support the findings of this study are available from the corresponding author upon reasonable request.
