# Supplementary material for: Elevated levels of sCD48 are inversely correlated with markers of disease activity in bullous pemphigoid
Source: Exp Dermatol. 2022 Oct 5;32(1):85–90. doi: 10.1111/exd.14679 (PMC9912975; doi:10.1111/exd.14679)
Supplement: Supplementary file 1 — Table S1 [file EXD-32-85-s001.docx]

**TableS1: Spearman’s correlation analysis of sCD48 with clinical and laboratory measures in subsets of BP patients^a^**

|  | BPDAI^a^ | BP180 IgG ^b^ | BP230 IgG ^b^ | BP180 IgE ^b^ | Total IgE ^b^ | neutrophils | lymphocytes | monocytes | eosinophils | basophils |
| --- | --- | --- | --- | --- | --- | --- | --- | --- | --- | --- |
| All BP (26)^d^ | -0.624  ***^e^ | -0.337 | -0.002  ns | -0.419  * | -0.043  ns | 0.036  ns | -0.274  ns | -0.029  ns | -0.461  * | -0.357  ns |
| BP180 IgG+ (24) | -0.564  *** | -0.219 | -0.091  ns | -0.555  ** | 0.214  ns | 0.070  ns | -0.304  ns | 0.070  ns | -0.449  * | -0.342  ns |
| BP180/BP230 IgG+ (12) | -0.666  * | -0.224  ns | -0.161  ns | -0.718  * | -0.133  ns | 0.168  ns | -0.309  ns | 0.239  ns | -0.501  ns | -0.484  ns |
| BP230 IgG+ (13) | -0.564  *** | -0.219  ns | -0.097  ns | -0.555  ** | -0.214  ns | 0.070  ns | -0.304  ns | 0.070  ns | -0.449  * | -0.342  ns |
| BP180 IgE+ (15) | -0.590  * | 0.032  ns | 0.046  ns | -0.007  ns | 0.254  ns | 0.140  ns | -0.336  ns | -0.197  ns | -0.029  ns | 0.271  ns |
| Total IgE >200 (16) | -0.670  *** | -0.253  ns | 0.124  ns | -0.319  ns | 0.188  ns | 0.016  ns | -0.449  ns | -0.091  ns | -0.160  ns | -0.113  ns |

^a^26 BP patients and 26 healthy controls

^b^Bullous Disease Area Index (BPDAI)

^c^BP180 and BP230 IgG, BP180 IgE and sCD48 were measured by ELISA. Total IgE was measured by electrochemiluminescence.

^d^subsets of BP patients

^e^*p≤0.05, ** p≤0.01, *** p≤0.001, ns = not significant.
